# A Rapid and Simple Procedure for the Establishment of Human Normal and Cancer Renal Primary Cell Cultures from Surgical Specimens

**DOI:** 10.1371/journal.pone.0019337

**Published:** 2011-05-04

**Authors:** Maria João Valente, Rui Henrique, Vera L. Costa, Carmen Jerónimo, Félix Carvalho, Maria L. Bastos, Paula Guedes de Pinho, Márcia Carvalho

**Affiliations:** 1 REQUIMTE - Laboratório de Toxicologia, Departamento de Ciências Biológicas, Faculdade de Farmácia, Universidade do Porto, Porto, Portugal; 2 Department of Pathology, Portuguese Oncology Institute-Porto, Porto, Portugal; 3 Department of Pathology and Molecular Immunology, Institute of Biomedical Sciences Abel Salazar (ICBAS), University of Porto, Porto, Portugal; 4 Cancer Epigenetics Group – Research Center, Portuguese Oncology Institute-Porto, Porto, Portugal; 5 Faculty of Health Sciences, University Fernando Pessoa, Porto, Portugal; The University of Hong Kong, China

## Abstract

The kidney is a target organ for the toxicity of several xenobiotics and is also highly susceptible to the development of malignant tumors. In both cases, *in vitro* studies provide insight to cellular damage, and represent adequate models to study either the mechanisms underlying the toxic effects of several nephrotoxicants or therapeutic approaches in renal cancer. The development of efficient methods for the establishment of human normal and tumor renal cell models is hence crucial. In this study, a technically simple and rapid protocol for the isolation and culture of human proximal tubular epithelial cells and human renal tumor cells from surgical specimens is presented. Tumor and normal tissues were processed by using the same methodology, based on mechanical disaggregation of tissue followed by enzymatic digestion and cell purification by sequential sieving. The overall procedure takes roughly one hour. The resulting cell preparations have excellent viabilities and yield. Establishment of primary cultures from all specimens was achieved successfully. The origin of primary cultured cells was established through morphological evaluation. Normal cells purity was confirmed by immunofluorescent staining and reverse transcription-polymerase chain reaction analysis for expression of specific markers.

## Introduction

Human proximal tubular epithelial cells (HPTEC) correspond to the major cell type in the human cortical tubulointerstitium and, most importantly, to the main target of a large number of xenobiotics, from drugs of abuse to antibiotics, antineoplastic agents, metals, and mycotoxins [1]–[5]. Primary cultures of HPTEC can provide a well-characterized *in vitro* model, phenotypically representative of HPTEC *in vivo*. This *in vitro* system is endorsed for investigation on kidney cell function, transport processes, and cellular mechanisms of proximal tubular injury by xenobiotics, without interference of other factors that are associated to *in vivo* experiments. For that purpose, it is essential to achieve highly enriched HPTEC preparations from kidney tissue. Several techniques have been described for isolation and culture of HPTEC. These methods have been based on time-consuming techniques like isopycnic centrifugation with Nycodenz or Percoll [6]–[9], or even complex microdissection protocols with or without enzymatic digestion [10], [11]. The major weaknesses of these methodologies include low yields and labor intense procedures.

In addition to xenobiotic-induced toxicity, the kidney is also susceptible to the development of benign (e.g., oncocytoma) or malignant (e.g., renal cell carcinoma, RCC) neoplasms. RCC comprises 85% of renal cancers in adults, and more than 3% of adult malignancies. With over 30,000 new cases diagnosed annually, it is the sixth leading cause of cancer-related death in the USA, being responsible for approximately 12,000 deaths per year [12], [13]. According to its histological appearance, RCC can be divided into subtypes: conventional or clear cell, papillary, chromophobe, and unclassifiable RCC [14], [15]. Clear cell RCC is the most common form of renal cancer. It is originated from the proximal tubular epithelium, and accounts for 80 to 85% of renal cell tumor [12], [15]. Papillary RCC is the second most usual subtype of kidney cancer, with a prevalence of roughly 10% of renal malignant tumors, and is characterized by tumor cells arranged in a papillary configuration [16], [17]. Chromophobe is an uncommon subtype of RCC, with a prevalence of approximately 5% of renal malignant tumors. As clear cell RCC, it develops in the renal cortex [18], [19].

RCC etiology is yet unidentified, developing either as a sporadic form or as a hereditary disease, and whatever the subtype is, it is described as highly resistant to conventional radio-, chemo- and immunotherapy regimens [12], [15], [20]. Therefore, the discovery of new strategies for therapeutic intervention remains a priority. In this regard, cell culture of human renal tumor cells (HRTC) has proven to be an adequate in vitro model for the study of therapeutic approaches in RCC [15], [21]–[23]. Moreover, alongside studies in tumor cell cultures, it is necessary to test the toxicity of potential therapeutic agents in the normal counterpart cells. Therefore, it is the main goal of this study to present a simple and rapid method for the establishment of human kidney primary cultures, both normal (HPTEC) and tumoral (HRTC), obtained from the same organ.

The procedure presented herein has been adapted from previously established methods [6], [9], [24]–[26] and used to process normal and tumor tissues. It is based on mechanical disaggregation of the tissue followed by enzymatic digestion and cell purification by sequential sieving. This technique allows the separation of a cellular fraction that is highly enriched in HPTEC or HRTC from respectively normal renal cortex and tumoral kidney tissue, with far higher yield and cell viability than other established isolation procedures. The overall procedure is technically simple, enabling its easy implementation in cell culture laboratories.

## Materials and Methods

### Materials

The following materials were obtained from GIBCO™ Invitrogen (Barcelona, Spain) unless stated otherwise.

**Figure 1:**
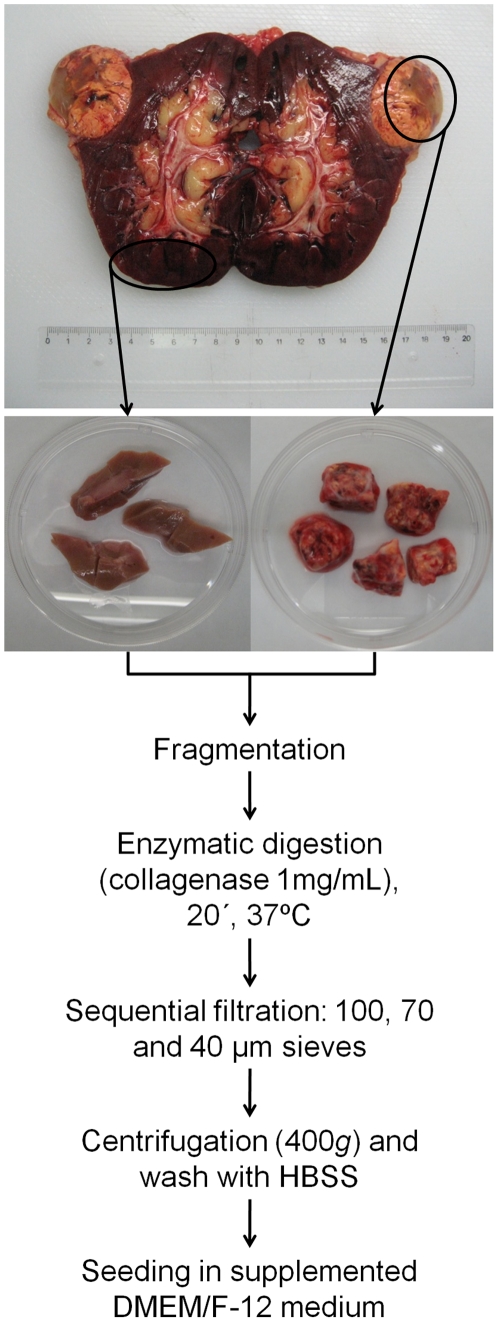
Schematic representation of the isolation procedure for HPTEC and HRTC.

1. Cell culture medium: Dulbecco's modified Eagle's medium with nutrient mixture F-12 (DMEM/F-12) and GlutaMAX-I™ supplemented with 10% heat-inactivated fetal bovine serum (FBS), penicillin/streptomycin (50 U/mL/50 µg/mL), fungizone (2.5 µg/mL), and human transferrin (5 µg/mL).
2. Hank's buffered salt solution (HBSS) without CaCl_2_ and MgCl_2_.
3. Collagenase solution: dissolve 50 mg of collagenase type 2, 315 U/mg (Worthington, Lakewood, NJ) in 25 mL non-supplemented culture medium and filter sterilized with 0.22 µm filter. Used fresh.
4. 0.05% Trypsin-EDTA solution.
5. Freezing solution: 90% FBS and 10% dimethylsulfoxide.
6. Incubation vessel: autoclaved 250 mL PYREX® glass-jacketed flask (Vidrolab 2, Gandra, Portugal) with a magnetic bar in it. The temperature of the collagenase solution in the reservoir is maintained at 37°C by circulating warm water through the jacket of the flask (Fig. 1).
7. Collagen coated flasks: 75-cm^2^ plastic culture flasks coated overnight at 37°C with a 40 µg/mL solution of collagen G from bovine calf skin (Biochrom AG, Berlin, Germany) in PBS.
8. Sterile cell strainers with sieve sizes of 100, 70, and 40 µm (BD Falcon™, BD Biosciences, USA).
9. 0.4% Trypan blue solution (Sigma Aldrich, St. Louis, MO).
10. AccuGENE® 1X PBS (Lonza Laboratories, Verviers, Belgium).
11. Immunocytochemical staining: mouse monoclonal anti-pan cytokeratin antibody, goat anti-mouse IgG-FITC antibody, and Hoechst 33258 (Sigma Aldrich, St. Louis, MO).
12. RT-PCR analysis: RNeasy Mini RNA isolation kit (Qiagen, USA) and RevertAid™ H Minus First Stand Synthesis Kit (Fermentas, Denmark).

### Ethics Statement

This study was approved by the Portuguese Oncology Institute-Porto Ethics Committee. All interviewed patients gave written informed consent.

### Tissue Collection

Human kidney tissue samples were obtained from a total of 6 patients (3 males and 3 females) undergoing radical nephrectomy for renal cell carcinoma at the Portuguese Oncology Institute-Porto (IPO-Porto). Patient mean age was 62±5 years old. Tissue samples (about 10 g each) were collected from areas macroscopically identified as normal (in the cortex) or tumoral immediately after the specimen extraction, by an expert uropathologist. The nature of those areas was subsequently confirmed by histopathological evaluation of mirror samples. Tissues were placed in separated sterile 50-mL tubes with ice-cold culture medium and then transferred to a cell culture laboratory on ice.

All specimens underwent subsequent routine tissue processing (formalin fixation and paraffin embedding). Histopathologic analysis of sections to assess the type of renal cancer, grading and staging was performed at the Department of Pathology, IPO-Porto.

### HPTEC and HRTC Isolation Protocol

Renal cell isolation took place within 30 minutes of renal tissue collection. All subsequent procedures were performed in a tissue-culture flow hood, under sterile conditions. To avoid cell cross-contamination, we recommend handling normal and tumor specimens in two individual tissue-culture hoods. If this is not possible, then, the following guidelines should be reinforced: only one surgical specimen should be used in a tissue-culture hood at any one time (during this time keep the other tissue on ice in a sterile container), the hood should be cleaned before the introduction of the other surgical specimen, and bottles or aliquots of medium should be dedicated for use with only normal or tumor cells.

1. In the laboratory culture cabinet, transfer the tissue to a 60-mm Petri dish. Using forceps and scissors, dissect off (i) the fibrous capsule and adjacent medulla from the cortical tissue and (ii) any fat, blood clots and connective tissues from tumor tissues.
2. Cut the tissue sample into small pieces with scalpels.
3. Transfer the tissue fragments to a sterile 50-mL centrifuge tube, rinse them vigorously with ice-cold HBSS (contains EGTA that loosen cell junctions via calcium chelating action) and decant the supernatant. Repeat this last step until the solution is clear of blood.
4. Pour off the supernatant, transfer the fragments to a clean 60-mm Petri dish and finely mince the tissue into approximately 1-mm^3^ pieces with scalpels.
5. Resuspend the small fragments in 25 mL pre-warmed non-supplemented culture medium and combine it with the collagenase solution (1 mg/mL final concentration) in the incubation vessel. Incubate for 20 minutes at 37°C with gentle stirring.
6. Pass the digested tissue onto the first sieve (100 µm) into a 50-mL centrifuge tube. The same procedure is then applied to the following sieves (70 and 40 µm). Sieving through the 100-µm sieve allows the removal of any undigested fibrous tissue, whereas the function of the 70 and 40-µm sieves is to remove contaminating tubular fragments and glomeruli, respectively.
7. Wash the sieved cells by centrifugation (400 *g*, 5 min at 4°C), and resuspend the pellet in HBSS. Repeat this process two more times, and then resuspend the cell pellet in culture medium with supplements. Determine cell number and viability in a Neubauer hemocytometer using the trypan blue solution.

Figure 1 presents a schematic representation of the overall procedure.

### Cell Culture Protocol

1. Seed the isolated cells on collagen-coated 75 cm^2^ culture flasks at a density of 5×10^4^ cells/cm^2^ and incubate in a humidified atmosphere of 95% air/5% CO_2_ at 37°C. (Note: The RPMI 1640 medium with supplements can be used alternatively to grow HRTC)
2. Change the medium 24 h after initial seeding and at 48 h intervals thereafter.
3. Allow the cells to grow until ∼80% confluence before they are subcultured or frozen.

When cultured as described above, the cells reach confluence in approximately 10–13 days after seeding.

### Cell Subculture Protocol

1. Remove the cell culture medium and wash the cell monolayer with either pre-warmed HBSS or PBS and 1 mL of trypsin-EDTA to weaken cell adhesion to the flasks surface.
2. Add enough trypsin-EDTA solution to cover the flask surface and incubate at 37°for 3 min. Observe the cells under an inverted optical microscope to ensure adequate cell detachment.
3. Terminate trypsin action by adding 10 mL of supplemented growth medium and resuspend the detached cells by repeatedly pipeting over the surface of the culture flask.
4. Transfer the resuspended cells to a centrifuge tube, rinse the flask with medium, and add the rinsed cells to a centrifuge tube. Seed the cell suspension into new 75-cm^2^ flasks at a 1∶3 subculture ratio.

Under these conditions, HPTEC beyond the first passage reach confluence in 3–5 days, and HRTC in 7 days.

### Cell Cryopreservation, Thawing and Replating Protocol

1. After trypsinization, transfer the detached cells to a centrifuge tube and pellet the cells by centrifugation (400 *g*, 5 min at 4°C).
2. Resuspend the pellet from each flask in 3 mL of freezing solution.
3. Tranfer 1 mL cell suspension to 2 mL cryovials and freeze immediately at −80°C.
4. To reseed the suspensions, cells are rapidly defrosted by adding pre-warmed supplemented medium and transferred to a 15-mL centrifuge tube.
5. Pellet the cells by centrifugation at 400 *g*, for 5 minutes.
6. The pellet is resuspended in warmed culture medium, and transferred to 75 cm^2^ flasks, one vial per flask.

HPTEC and HRTC are successfully cryopreserved from both isolated cells and cell suspensions obtained after trypsinisation, maintaining a normal growth after defrosting.

### Morphological Evaluation

Monolayer cultures derived from all 12 primary isolates (6 normal, 4 clear cell RCC and 2 chromophobe RCC) and the corresponding first three passages were examined by light microscopy under an inverted optical microscope. An immortalized proximal tubule epithelial cell line derived from normal adult human kidney, HK-2 cell line (ATCC, CRL-2190), was also examined for comparison.

### Immunocytochemical Staining for Cytokeratin

To investigate the epithelial origin of obtained normal and tumor renal cells, the reactivity to anti-cytokeratin antibody was assessed in suspensions from passages 1 to 3. Two immortalized cell lines derived from normal (HK-2) and tumor (A-498) human kidney were used as positive controls.

Briefly, HPTEC or HRTC were grown to about semiconfluence on 24-well plates (initial density: 1.0×10^5^ cells/well), washed with sterile PBS, fixed for 20 minutes in 4% *p-*formaldehyde, and permeabilized for 5 minutes in 1% Triton X-100 solution. After blocking with a 1% bovine serum albumin, 0.4% Triton X-100 and 4% sodium azide mixture, the cells were incubated overnight at 4°C with mouse monoclonal anti-pan cytokeratin antibody (1∶100), and then with goat anti-mouse IgG-FITC antibody (1∶100) for 2 hours at room temperature. After rinsing with PBS, nuclei were counterstained with 5 µg/mL Hoechst 33258 for 5 minutes and then washed with PBS again. Images were captured using a fluorescence microscope (Nikon, Eclipse E400). Appropriate negative controls were included to guarantee positive antibody reactivity.

### Reverse Transcriptase-PCR (RT-PCR) Analysis for Specific Markers

Suspensions (about 3.0×10^6^ cells each) of first passage normal cells from 4 donors were analyzed by RT-PCR to confirm the origin and purity of the isolates. Hence, the mRNA expression of three characteristic proteins was evaluated: uromodulin (distal tubular cells), aquaporin 3 (collector duct cells) and aminopeptidase A (proximal tubular cells). Briefly, RNA was extracted from all samples with RNeasy Mini RNA isolation kit, and concentrations were determined using a ND-1000 Nanodrop (NanoDrop Technologies, Wilmington, USA). cDNA was prepared from RNA using the RevertAid™ H Minus First Stand Synthesis Kit and then amplified by RT-PCR using the previously published primers sequences and respective annealing conditions [27]. Glyceraldehyde 3-phosphate dehydrogenase (GAPDH) was used as the housekeeping gene, a mixture of renal cells as the positive control for all proteins analyzed, and water as the negative control. RT-PCR products were loaded onto nondenaturing 2% agarose gels, stained with ethidium bromide and visualized under an UV transilluminator.

## Results

### Histopathology

**Figure 2:**
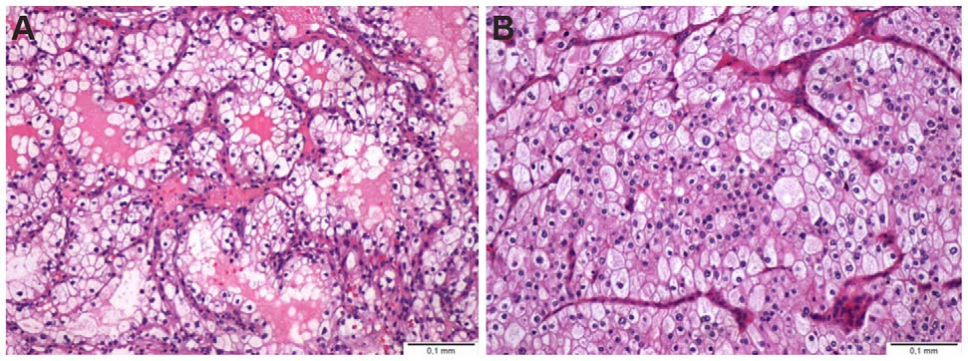
Representative histological images of renal cell carcinoma. (A) Clear cell renal cell carcinoma, and (B) chromophobe renal cell carcinoma.

The HRTC cultures were derived from RCC clear cell (4 cases) or chromophobe (2 cases) subtypes. The clear cell RCC cases were composed by cells in acinar or alveolar arrangement, which displayed the characteristic optically clear cytoplasm with distinct cell membranes, variable nuclear size and conspicuousness of the nucleoli (Fig. 2A). The chromophobe RCC cases were characterized by large cells, with slightly eosinophilic cytoplasm, prominent cell membranes and irregularly shaped nuclei (Fig. 2B). Both cases stained diffusely in the cytoplasm with Hale's colloidal iron stain.

### Characteristics of Primary Cell Cultures

Table 1 summarizes the main characteristics of each donor, and the corresponding isolates. The method provides preparations of renal cells with excellent cell viabilities (96.5±1.4 % and 89.7±4.1 % for normal and tumor renal cells, respectively) and yields (18.7±6.9×10^6^ HPTEC/g of cortical tissue and 3.1±4.7×10^6^ HRTC/g of tumoral tissue). Time spent, from the initial collection of the kidney specimens to the seeding of isolates, took approximately 1 hour.

**Table 1:** Donors characteristics and respective isolates parameters.

Donor	Age	Sex	Type of RCC	Cell viability (%)		Cell recovery (cells/gram of tissue)	
				HPTEC	HRTC	HPTEC	HRTC
1	72	Male	Clear cell	96.9	91.6	23.7×10^6^	12.2×10^6^
2	61	Female	Clear cell	98.3	84.6	29.9×10^6^	5.9×10^5^
3	61	Female	Chromophobe	95.6	90.5	14.8×10^6^	7.3×10^5^
4	62	Female	Chromophobe	96.1	92.3	18.3×10^6^	4.2×10^6^
5	56	Male	Clear cell	94.5	84.6	11.9×10^6^	2.2×10^5^
6	61	Male	Clear cell	97.6	94.4	13.5×10^6^	7.2×10^5^

The establishment of primary cultures was successfully attained from all twelve tissue specimens (6 from normal cortex plus 4 from clear cell RCC and 2 from chromophobe RCC). All were maintained in culture for a minimum of three passages. Primary isolated cells, normal and tumor-derived, reached confluence within approximately 10 days. After the first passage, normal kidney epithelial cells exhibited a greater propensity to proliferate *in vitro* than tumor cells. All primary cultures (first and subcultures) were free of fibroblastic overgrowth.

**Figure 3:**
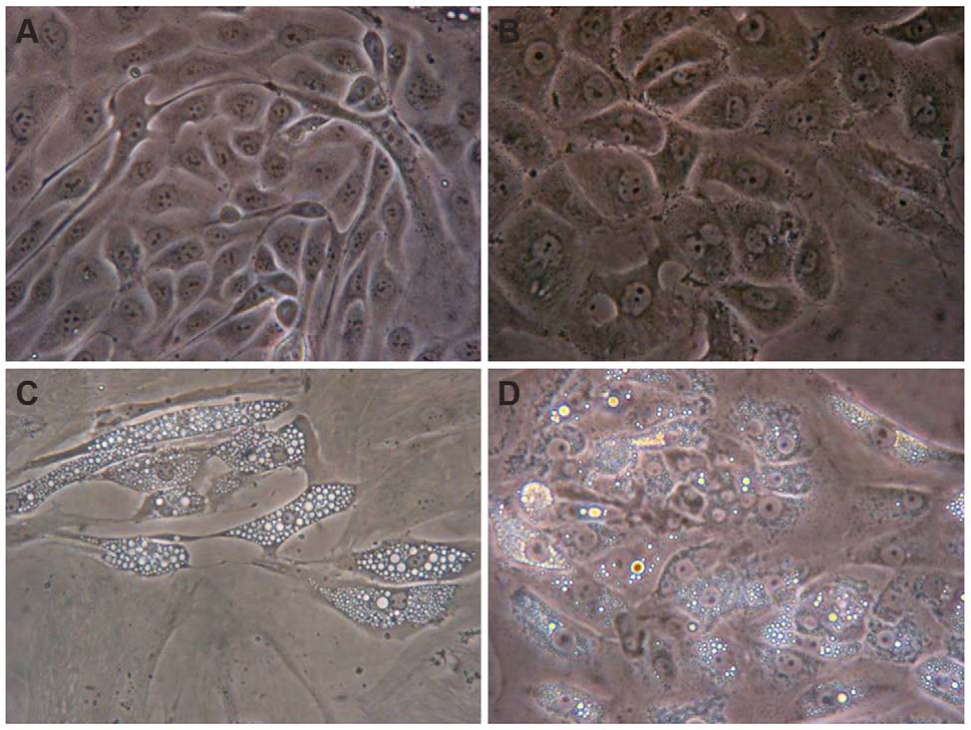
Representative morphology of confluent monolayers. (A) Primary HPTEC, (B) HK-2 cell line, (C) primary clear cell RCC, and (D) primary chromophobe RCC. Magnification: 400x.

Under phase-contrast microscopy, confluent primary cultures of normal renal cells showed a very homogeneous morphology. HPTEC in culture exhibit a cobblestone appearance (Fig. 3A), characteristic of epithelial cells like the HK-2 cells (Fig. 3B). Formation of typical domes (hemicysts) was visible when the HPTEC cultures become highly confluent, indicative of transepithelial solute transport by the monolayers.

**Figure 4:**
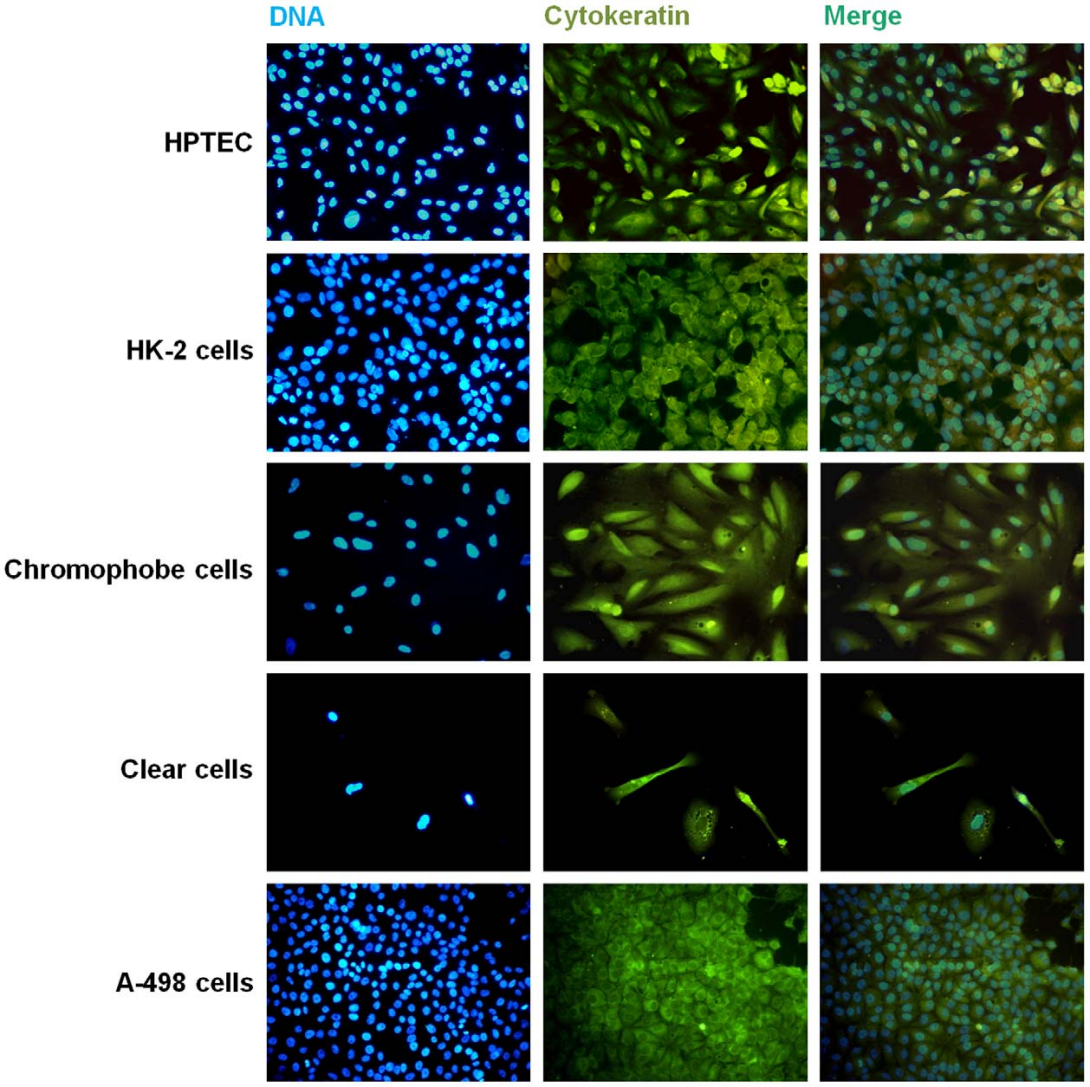
Fluorescence microscopy images from immunocytochemical staining for cytokeratin. Hoechst 33258, anti-cytokeratin antibody staining, and merged images with double staining of primary cultured HPTEC, clear cell and chromophobe RCC cells. HK-2 and A-498 cells were used as control of human normal and tumor epithelial kidney cells, respectively. Magnification: 200x.

HRTC were efficiently isolated from clear cell and chromophobe RCC and characterized by their flattened polygonal morphology and presence of lipid vesicles in the cytoplasm. These tumor subtypes could be distinguished by the number and size of the vesicles: clear cells present the entire cytoplasm occupied by multidimensional vesicles (Fig. 3C), while in chromophobe cells the vesicles appeared in smaller amounts and dimension, and the cytoplasm is not “empty” as in clear cell RCC (Fig. 3D). Light microscopic appearance of primary isolates (normal and tumoral) and the following three passages was not appreciably different. Cytokeratin was expressed in all RCC primary cultures (Fig. 4), bearing out their epithelial origin.

**Figure 5:**
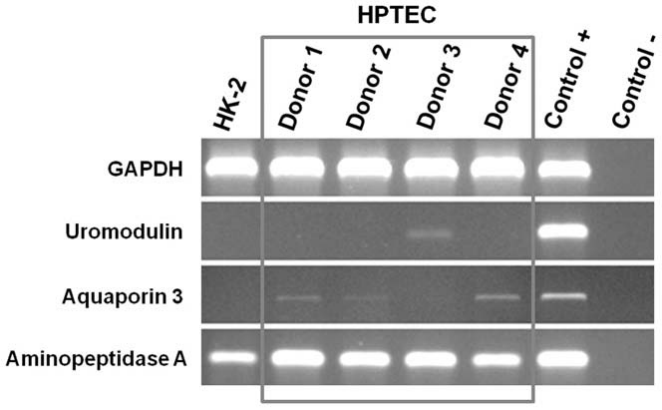
RT-PCR analysis. Uromodulin (distal tubule marker), aquaporin 3 (collecting duct marker), and aminopeptidase A (proximal tubule marker) in primary cultured HPTEC derived from 4 donors and HK-2 cells. RNA isolated from a mixture of renal cells was used as a positive control and water as a negative control. Expression of glyceraldehydes-3-phosphate dehydrogenase (GAPDH) was used as a house-keeping gene.

To further characterize the proximal tubule cells in primary culture, expression of specific marker proteins was evaluated by immunocytochemical staining with mouse monoclonal antibody against human cytokeratin and RT-PCR analysis for uromodulin, aquaporin 3, and aminopeptidase A. These cells expressed cytokeratin uniformly, which was similar to that of HK-2 cells (Fig. 4), thus confirming its epithelial nature. RT-PCR analysis of HPTEC primary cultures from 4 patients was performed. All four isolates retained molecular properties of HPTEC, showing high expression of aminopeptidase A, the characteristic protein of proximal tubular cells (Fig. 5). Furthermore, the cells had very week or lacked the expression of the distal tubular and collecting duct markers, uromodulin and aquaporin 3, respectively.

## Discussion

Several *in vitro* kidney preparations have been used for physiologic and toxicologic studies, including isolated perfused kidney, renal slices, nephron segments, cell lines, and primary cultures [28]. Among these, cell culture offers the advantages of availability of greater number of cells and facility to perform multiple and repeated experiments over long time periods. The use of established cell lines for toxicologic studies can pose many limitations when compared to primary cultures. In fact, the value of toxicologic studies performed on essentially transformed cells may be questionable. It is well known that immortalized cell lines undergo dedifferentiation (i.e., the loss of the original cell specificity) as a result of prolonged passage *in vitro* [29], and possess characteristics that are not present in their tissue of origin due to their immortalization. Therefore, an altered cellular response to toxicants may be seen. In contrast, primary cultures retain many of phenotypic characteristics of the original tissue, including normal physiological functions, and, therefore, can be highly relevant models for gene discovery, target validation, drug testing, and development of biomarkers. In addition, utilizing multiple donors for samples of tissue to generate independent isolates of primary cells allows researchers to demonstrate consistent responses among individuals.

Although several authors have previously reported methods for isolation and primary culture of HPTEC [6], [9], [11] and HRTC [12], [14], widespread application of these human culture preparations has been hampered by modest yields and impaired cell viability.

In this study, we describe an optimized method for establishing human primary cell cultures from both normal and tumor tissues, harvested from the patient's specimen immediately after radical nephrectomy procedure. Of note is that specimen collection as soon after resection as possible is an important factor for obtaining cell suspensions with high viability as there is a clear decline in the amount of successfully cultured material the longer the tissue remains *ex vivo* [24].

Given that proximal tubules location is confined entirely to the renal cortex, cultures were established by using cells isolated by progressive enzymatic dissociation from the extreme outer cortex of the normal human kidney. Mincing the specimen into small pieces allows good removal of red blood cells during initial washing steps and maximizes enzymatic digestion. Appropriate choice of enzyme, incubation time, temperature, and concentration for optimal digestion are required to obtain the best tissue dissociation without excessive destruction. Collagenase digestion is known to be less harmful to epithelial cells than is digestion with other enzymes (as trypsin) and under conditions set out in our protocol gives cell suspensions with higher yields and viabilities than those established for previous isolation procedures. Sequential filtration of collagenase digest through a series of sieves with decreasing mesh sizes removes tubular fragments and glomeruli, and leaves material that yields outgrowth of renal epithelial cells with a proximal phenotype most probably due to their high proliferative potential. In addition, the first medium change, 24 h after plating, minimizes attachment of other non-epithelial cells.

A procedure should be relatively simple and fast to minimize membrane damage during isolation and time required before the cells can be used in experiments. This is achieved in our method as it does not include time-consuming steps like Percoll density gradient centrifugation [6], [9], or complex microdissection [10], [11] or immunomagnetic techniques [30]. The procedure takes nearly 1 hour to perform, thus enabling a faster establishment of primary cell cultures when compared to previous HPTEC or HRTC culture protocols.

The establishment of primary cultures was successfully attained from all surgical specimens. A total of 12 different renal primary cultures were obtained. Among these cultures, 6 were from normal cortex, 4 from clear cell RCC and 2 from chromophobe RCC. In our procedure, the tumor primary cultures take longer to reach the subconfluence beyond the first split than the normal cortex primary cultures. The normal cortex primary cultures presented a homogeneous morphology, while the primary tumor cultures had a more heterogeneous morphology. The normal renal cell cultures displayed an epithelial morphology that was consistent with reported characteristics for HPTEC [9], [24], [25]. Optical microscopic analysis up to the third passage showed that this differentiated morphology did not alter on subculture. We did not try to check the life span of primary normal or tumor cultures because it is well-known that a variation of phenotype occurs at higher passages [26], [29], [30]. The growth pattern and morphology of HK-2 cells did not differ from that of primary cultured HPTEC, as assessed by light microscopy. Importantly, fibroblast contamination was not observed in our short-term primary cultures, but may be a problem after longer primary culture or further subcultures. To overcome this problem, it has been recommended the substitution of L-valine by D-valine and L-arginine by L-ornithine in HPTEC medium [6] or the use of hormone-supplemented serum-free culture medium [9].

The presence of specific markers was established to characterize the HPTEC primary cultures. In particular, the epithelial nature of the HPTEC cultures was confirmed by immunocytochemistry with positive staining for cytokeratin and the proximal tubular origin by RT-PCR with clear expression of the proximal tubule brush border enzyme aminopeptidase A. Furthermore, these cells showed very weak or absent expression of the distal tubular and collecting duct markers, uromodulin and aquaporin 3, respectively. These results indicate that our primary HPTEC cultures are originated from proximal tubule cells, without significant contamination of cells from other parts of the nephron. Thus, our method enables to isolate and grow highly differentiated primary cultures, which express specific proximal tubular proteins.

The cytological homogeneity of our HPTEC primary cultures was evaluated by immunocytochemical staining for cytokeratin up to the third passage, and in all instances the reactions were homogeneous in intensity and had identical profiles.

Cell characterization performed in this study corroborates other studies showing that HPTEC cultures retain the differentiated characteristics of PTC for up to three passages [9], [31]. This *in vitro* model may be employed for studies concerning renal injury, including xenobiotic-induced toxicity in HPTEC, therefore eliminating the need for extrapolation from animal cell cultures and more representative of renal cells *in vivo* than established kidney cell lines.

Of note is that these renal cells of proximal tubule may be grown in permeable membrane filter supports, resulting in cells that morphologically and functionally better represent their *in vivo* counterparts. Cells cultured in these membrane supports are used to study proximal transport systems, as well as the effect of different xenobiotics at both the apical and basolateral sides of the proximal tubular cell [32].

The procedure was also efficiently applied to the isolation of HRTC from two morphologically and genetically distinct subtypes of RCC, namely clear cell and chromophobe RCC. Both types develop from human renal cortical cells. However, clear cell RCCs are believed to originate from the epithelium of proximal convoluted tubules, whereas chromophobe RCCs seem to derive from the cortical segment of the collecting duct [33]. While primary cultures of normal epithelium cells of proximal origin present a very homogeneous morphology, significant morphological heterogeneity among clear cell RCC cultures were noted. This result is in accordance with previous reports showing variations in their size and shape [34]. The cells presented the expected histological characteristics described in previous studies [18], [19], [35].

The method proved to be technically simpler, faster and with a higher rate of success for the establishment of tumor primary cultures from surgical specimens than the established for previous isolation procedures [12], [14]. Primary cultures from RCC cancers are useful tools to study the biochemical and molecular changes associated with the neoplastic status. In addition, the development of RCC cultures, alongside with their corresponding normal counterparts, may yield a more realistic model for the development and testing of new therapeutic modalities.

In conclusion, we present a useful and simple method for successful establishment of primary cultures derived from fresh human normal cortical and tumor tissues. It combines different techniques already described in the literature, and results in an optimized isolation method, yielding cell preparations with excellent viability and recovery. Moreover, it is less labor intense and time-consuming than previous isolation procedures.

The characterization of primary cultures indicated their proximal tubule or RCC origin and demonstrated that these cultures at low passages preserve *in vivo* properties, and represent, therefore, valuable *in vitro* models for use in physiology, pharmacology, toxicology, and oncobiology studies.
